# Coupling Analysis of Flexoelectric Effect on Functionally Graded Piezoelectric Cantilever Nanobeams

**DOI:** 10.3390/mi12060595

**Published:** 2021-05-21

**Authors:** Yuhang Chen, Maomao Zhang, Yaxuan Su, Zhidong Zhou

**Affiliations:** 1 Fujian Provincial Key Laboratory of Advanced Materials, College of Materials, Xiamen University, Xiamen 361005, China; 20720181150006@stu.xmu.edu.cn (Y.C.); 18138803875@163.com (M.Z.); 2 Chengyi University College, Jimei University, Xiamen 361021, China; suyaxuan@jmu.edu.cn

**Keywords:** flexoelectric effect, functionally graded beam, generalized variational principle, piezoelectricity, induced electric potential

## Abstract

The flexoelectric effect has a significant influence on the electro-mechanical coupling of micro-nano devices. This paper studies the mechanical and electrical properties of functionally graded flexo-piezoelectric beams under different electrical boundary conditions. The generalized variational principle and Euler–Bernoulli beam theory are employed to deduce the governing equations and corresponding electro-mechanical boundary conditions of the beam model. The deflection and induced electric potential are given as analytical expressions for the functionally graded cantilever beam. The numerical results show that the flexoelectric effect, piezoelectric effect, and gradient distribution have considerable influences on the electro-mechanical performance of the functionally graded beams. Moreover, the nonuniform piezoelectricity and polarization direction will play a leading role in the induced electric potential at a large scale. The flexoelectric effect will dominate the induced electric potential as the beam thickness decreases. This work provides helpful guidance to resolve the application of flexoelectric and piezoelectric effects in functionally graded materials, especially on micro-nano devices.

## 1. Introduction

With the development of micro-nano technology, smart materials have received widespread attention at the nanoscale. For the micro-nano devices, the flexoelectric effect on nano-piezoelectric beams must be considered in the study of electro-mechanical coupling. Piezoelectric and flexoelectric effects are ubiquitous in a wide variety of materials, including artificial and natural materials [1,2,3,4]. The conventional electro-mechanical coupling between the electric polarization and the uniform strain is unique for noncentrosymmetric crystals, such as piezoelectric materials [5]. However, the presence of the strain gradient or nonuniform strain field can locally break the inversion symmetry and induce an electric polarization even in crystalline centrosymmetric dielectrics. This spontaneous electric polarization induced by the strain gradient is referred to as flexoelectricity, which is proportional to both the flexoelectric coefficient and the magnitude of the strain gradient. The flexoelectricity may lead to strong size-dependent properties at the nanoscale. Therefore, it is necessary to consider the flexoelectric effect in analyzing the electro-mechanical coupling of dielectrics at the nanoscale.

Recently, a series of studies to discuss the flexoelectricity of ferroelectric thin films, polymers, liquid crystals, and living membranes have been reported. Majdoub et al. [6] used molecular dynamics and linear piezoelectric theory to analyze the piezoelectric nanobeam under the nonuniform strain condition. Applying a variational principle for dielectrics by incorporating the flexoelectricity, electrostatic force, and surface effect, Hu and Shen [7,8] developed the general governing equations of flexoelectric materials. Yan and Jiang [9] investigated the electroelastic responses of the piezoelectric nanobeams with the flexoelectric effect, in which the surface effect has been taken into consideration. Zhou et al. [10] presented an electro-mechanical method to analyze the flexoelectric beams with three different electrical boundary conditions, in which the induced electric potential has been discussed. Su et al. [11] studied the influence of the flexoelectric and piezoelectric effects on electro-mechanical coupling responses of the bilayer piezo-flexoelectric nanobeam based on the strain gradient elasticity. The results show that the flexoelectric and strain gradient elastic effects dominate the electro-mechanical response at the nanoscale, while the piezoelectric effect is the opposite. Recently, Su and Zhou [12] investigated the nonlocal effect on the flexoelectric beams based on the generalized Hamilton’s variational principle, in which the induced electric potential of the flexoelectric sensor is dependent on the category of applied loads.

Up to now, some results about the flexoelectric effect on the static bending and vibration behaviors of monolayer or multi-layer nanobeams have been obtained. However, studies on the flexoelectric effect are very limited, especially in improving the flexoelectric effect through structural design or the uneven distribution of materials. In view of the limitations of forming a large nonuniform strain or strain gradient and the inconvenience of the practical applications of irregular geometric shapes in ordinary dielectric materials, introducing functionally graded materials (FGMs) and multi-layer structure materials to improve the material properties is a very useful and wise method [13,14,15,16]. Regarding functionally graded piezoelectric materials, many studies have been carried out [17,18]. With a continuum model self-consistently treating piezo-flexoelectricity, Abdollahi and Arias [19] studied the interplay between piezoelectricity and flexoelectricity in bimorph sensors and actuators via the smooth meshfree method. Their results showed that flexoelectricity might enhance or reduce the effective piezoelectric effect depending on the device scale. Mbarki et al. [20] researched a combination of flexoelectricity and simple functional gradient to acquire high-temperature electro-mechanical coupling in a thin film. Chu et al. [21] investigated the flexoelectric effect on the bending and vibration responses of functionally graded piezoelectric nanobeams based on a modified strain gradient theory. Xiang et al. [22] studied the electro-elastic response on the bending behavior of a functionally graded elastic beam with consideration of the flexoelectric effect. They only explained the role of the flexoelectric effect in functionally graded beams. Chen et al. [23] analyzed the effect of flexoelectricity on the free vibration of functionally graded porous piezoelectric sandwich nanobeams reinforced by graphene platelets. They found that the vibration behavior of the nanobeam would be effectively influenced by the flexoelectricity, porosity, and graphene platelets. However, it is not clear how the coupling of flexoelectric and piezoelectric effects acts on the electro-mechanical responses of FGM beams. In functionally graded piezoelectric materials, the effective flexoelectric coefficient caused by piezoelectricity is functionally identical to intrinsic flexoelectricity [24]. Thus, it is very important and beneficial to analyze and discuss the coupling effect of piezoelectricity and flexoelectricity in the FGM beams.

In this paper, the objective is to deal with the electro-mechanical responses of the FGM piezoelectric beam considering the flexoelectric effect. Based on the linear piezo-flexoelectric model, the equilibrium equations of FGM beams and corresponding general mechanical boundary conditions are derived by the generalized variational method. The influences of the coupling between flexo-piezoelectricity and the gradient parameter on the deflection, induced electric potential, and stress distribution are presented graphically, analyzed, and discussed.

## 2. Formulation of Flexoelectric Materials

Based on the piezoelectric linear theory, considering the coupling effect of the strain gradient and the electric field, the electric Gibbs free energy density function U of the flexo-piezoelectric material can be expressed as [7,10,25]

(1) $$U=-\frac{1}{2}a_{kl}E{\;}_{k}E_{l}+\frac{1}{2}c_{ijkl}\varepsilon_{ij}\varepsilon_{kl}-e_{ijk}E_{i}\varepsilon_{jk}-\mu_{ijkl}E_{i}\varepsilon_{jk,l}$$

where $a_{kl}$ and $c_{ijkl}$ are the second-order dielectric coefficient and the fourth-order elastic coefficient, respectively. $e_{ijk}$ is the third-order piezoelectric coefficient, and $\mu_{ijkl}$ is the fourth-order flexoelectric coefficient. $\varepsilon_{ij}$, $\varepsilon_{jk,l}$ and $E_{i}$ are the strain tensor, the strain gradient tensor and electric field, respectively.

Under the framework of linear deformation theory, from Equation (1), the constitutive equations of the flexo-piezoelectric materials can be reduced to

(2) $$\sigma_{ij}=\frac{\partial U}{\partial \varepsilon_{ij}}=c_{ijkl}\varepsilon_{ij}-e_{ijk}E_{k}$$

(3) $$\sigma_{ijk}=\frac{\partial U}{\partial \varepsilon_{ij,k}}=-\mu_{ijkl}E_{l}\;$$

(4) $$D_{i}=-\frac{\partial U}{\partial E_{i}}=a_{ij}E_{j}+e_{ijk}\varepsilon_{jk}+\mu_{ijkl}\varepsilon_{jk,l}$$

where $\sigma_{ij}$, $\sigma_{ijk}$ and $D_{i}$ are the Cauchy stress tensor, the higher-order stress tensor and electric displacement vector, respectively. In classical electrodynamics, $D_{i},$ $E_{i}$ and polarization $P_{i}$ have the relationships as follow

(5) $$D_{i}=\varepsilon_{0}E_{i}+P_{i}$$

(6) $$P_{i}=\chi_{ij}E_{j}+e_{ijk}\varepsilon_{jk}+\mu_{ijkl}\varepsilon_{jk,l}$$

where $\chi_{ij}$ denotes electric susceptibility, and $\varepsilon_{0}$ is the electric permittivity of the vacuum. Then, from Equations (2)–(4), we can obtain

(7) $$U=\frac{1}{2}\sigma_{ij}\varepsilon_{ij}+\frac{1}{2}e_{ijk}\varepsilon_{ij,k}-\frac{1}{2}D_{i}E_{i}$$

Considering the work performed by external forces, the expression of the total enthalpy of flexo-piezoelectric materials [10,26]

(8) $$H=∭Udv-∯t_{i}u_{i}ds-∯r_{i}v_{i}ds+∯\varpi \phi ds$$

where ϖ is the surface charge density, ϕ is the surface potential, $t_{i}$ is the surface mechanical force, $r_{i}$ and $v_{i}$ are the higher-order tension and the corresponding normal derivative of displacement on the surface, respectively.

## 3. Beam Models of Functionally Graded Materials

**Figure 1:**
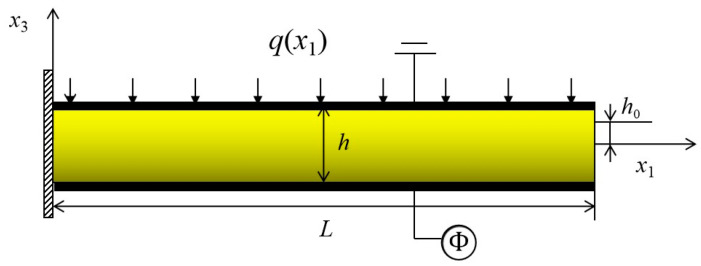
The geometry and coordinates of an FGM piezoelectric cantilever beam.

In the following, the functionally graded piezoelectric cantilever nanobeam has been considered. The material changes continuously from one material to another along the thickness direction. An FGM piezoelectric nanobeam with length *L*, width *b* and thickness *h* is shown in Figure 1. BaTiO_3_ (BTO) is a perovskite piezoelectric ceramic, which has outstanding electro-mechanical performance and large flexoelectric parameters. However, BTO is too stiff to generate a large deformation or large stain gradient, which is very important for the flexoelectric structures [1,3]. PVDF has very low stiffness and is easy to deform, in which a large strain gradient would exist in flexoelectric structures [3]. Combining the advantages of BTO and PVDF, FGM piezoelectric structures could have greater flexoelectric coefficients and considerable strain gradient. Hence, the nanobeam is made from a mixture of two isotropic linear elastic constituents BaTiO_3_ and PVDF, and the top and bottom surfaces of the beam are covered with electrodes. It is assumed that the top surface ($x_{3}=h/2$) of the FGM nanobeam is PVDF-rich while the bottom surface ($x_{3}=-h/2$) is BaTiO_3_ -rich. The bottom surface electrode undergoes a change of electric potential as a result of mechanical deformation or prescribes an external voltage, and the top electrode is grounded.

All material properties are assumed to satisfy the unified exponential law distribution

(9) $$M\left(x_{3}\right)=M_{0}f\left(x_{3}\right)$$

where $f\left(x_{3}\right)$ is an exponential function of $x_{3}$, which can represent the gradient type of material property change. M expresses all material properties of FGM piezoelectric beam, $M_{0}$ denotes the corresponding material property at the bottom surface $x_{3}=-h/2$. Hence, we have the gradient type as follows [27]

(10) $$M\left(x_{3}\right)=M_{0}e^{\alpha \left(\frac{2x_{3}+h}{2h}\right)}$$

where $\alpha =\ln\left(\frac{M\left(\frac{h}{2}\right)}{M_{0}}\right)$ is a gradient index, which can be determined by the graded properties in the physical beam. Through Equation (10), we can obtain the dielectric coefficient $a\left(x_{3}\right)$, piezoelectric coefficient $e\left(x_{3}\right),$ flexoelectric coefficient $\mu \left(x_{3}\right)$, and elastic coefficient $c\left(x_{3}\right)$ of the graded beam

(11) $$\{\begin{array}{c} c\left(x_{3}\right)=c_{1111}e^{\alpha_{1}\left(\frac{2x_{3}+h}{2h}\right)}\\ e\left(x_{3}\right)=e_{311}e^{\alpha_{2}\left(\frac{2x_{3}+h}{2h}\right)}\\ \mu \left(x_{3}\right)=\mu_{3113}e^{\alpha_{3}\left(\frac{2x_{3}+h}{2h}\right)}\\ a\left(x_{3}\right)=a_{33}e^{\alpha_{4}\left(\frac{2x_{3}+h}{2h}\right)} \end{array}$$

For simplicity, the gradient indexes of $a\left(x_{3}\right)$, $e\left(x_{3}\right)$ and $\mu \left(x_{3}\right)$ are set as the same constant α. According to the Euler–Bernoulli beam theory, the corresponding strain and strain gradient of the graded beam can be expressed as

(12) $$\varepsilon_{11}=-\left(x_{3}-h_{0}\right)\frac{d^{2}w}{dx_{1}^{2}}$$

(13) $$\varepsilon_{11.3}=-\frac{d^{2}w}{dx_{1}^{2}}$$

(14) $$\varepsilon_{11.1}=-\left(x_{3}-h_{0}\right)\frac{d^{2}w}{dx_{1}^{2}}$$

where w is the deflection of the beam in the $x_{3}$ direction, $h_{0}$ denotes the deviation between the physical neutral surface and the geometric mid-surface. In this paper, the axial strain gradient $\varepsilon_{11.1}$ is much smaller than the transverse strain gradient $\varepsilon_{11.3}$ in a slender beam, so the gradient in the axial direction is not considered. Here it should be pointed out that the physical neutral surface of the FGM nanobeam coincides with the geometric mid-surface in homogeneous materials. However, the symmetry breaking of FGMs influences the position of the physical neutral surface significantly. The position of the physical neutral surface can be determined using the following formula [28]

(15) $$h_{0}=\frac{\int_{A}c\left(x_{3}\right)x_{3}dA}{\int_{A}c\left(x_{3}\right)dA}$$

where A is the cross-section area of FGM cantilever nanobeam. The electric field is predominant in the thickness direction, while the electric field in the length direction is negligible in the slender beam. We substitute Equations (12) and (14) into Equations (2)–(4) to gain the expressions of stress, higher-order stress, and electric displacement of graded beams

(16) $$\sigma_{11}=c\left(x_{3}\right)\varepsilon_{11}-e\left(x_{3}\right)E_{3}$$

(17) $$\sigma_{113}=-\mu \left(x_{3}\right)E_{3}$$

(18) $$D_{3}=a\left(x_{3}\right)E_{3}+e\left(x_{3}\right)\varepsilon_{11}+\mu \left(x_{3}\right)\varepsilon_{11.3}$$

The electric field $E_{3}$ in the functionally graded beam can be expressed as the gradient of the internal electric potential Φ along the thickness direction. Without free body charge in the nanobeam, Gauss’s law of electrostatics is used to obtain the equation about electric potential

(19) $$\frac{\partial^{2}\Phi }{\partial x_{3}^{2}}=-\frac{\alpha }{h}\frac{\partial \Phi }{\partial x_{3}}-x_{3}\frac{\alpha }{h}\frac{e_{311}}{a_{33}}\frac{d^{2}w}{dx_{1}^{2}}-\left[\left(1-\frac{\alpha h_{0}}{h}\right)\frac{e_{311}}{a_{33}}+\frac{\alpha }{h}\frac{\mu_{3113}}{a_{33}}\right]\frac{d^{2}w}{dx_{1}^{2}}$$

In Figure 1, the electric potentials on the top and bottom surfaces of the nanobeam are

(20) $$\{\begin{array}{c} \Phi \left(\frac{h}{2}\right)=0\\ \Phi \left(-\frac{h}{2}\right)=\phi \left(x_{1}\right) \end{array}$$

where $\phi \left(x_{1}\right)$ is an applying external voltage or the inducing electric potential as a result of mechanical deformation. Solving Equation (19), we obtain

(21) $$\Phi \left(x_{1},x_{3}\right)=r\left(x_{3}\right)\phi \left(x_{1}\right)+\left[nhr\left(x_{3}\right)+mx_{3}^{2}+nx_{3}\right]\frac{d^{2}w}{dx_{1}^{2}}+C\left(x_{1}\right)$$

where $n=\frac{h_{0}e_{311}-\mu_{3113}}{a_{33}},\;m=-\frac{e_{311}}{2a_{33}}$, $r\left(x_{3}\right)=e^{-\frac{\alpha x_{3}}{h}}/\left(e^{\frac{\alpha }{2}}-e^{-\frac{\alpha }{2}}\right)$ and $C\left(x_{1}\right)$ is a function of $x_{1}$. Therefore, the electric field can be obtained

(22) $$E_{3}=\frac{\alpha }{h}r\left(x_{3}\right)\phi \left(x_{1}\right)+\left[\alpha nr\left(x_{3}\right)-2mx_{3}-n\right]\frac{d^{2}w}{dx_{1}^{2}}$$

From Equations (16)–(18), we obtain

(23) $$\sigma_{11}=-\left[c\left(x_{3}\right)\left(x_{3}-h_{0}\right)+e\left(x_{3}\right)\left(\alpha nr\left(x_{3}\right)-2mx_{3}-n\right)\right]\frac{d^{2}w}{dx_{1}^{2}}\;-\frac{\alpha e\left(x_{3}\right)\phi \left(x_{1}\right)}{h}r\left(x_{3}\right)$$

(24) $$\sigma_{113}=-\mu \left(x_{3}\right)\frac{\alpha r\left(x_{3}\right)\phi \left(x_{1}\right)}{h}-\mu \left(x_{3}\right)\left(\alpha nr\left(x_{3}\right)-2mx_{3}-n\right)\frac{d^{2}w}{dx_{1}^{2}}$$

(25) $$D_{3}=a\left(x_{3}\right)\frac{\alpha r\left(x_{3}\right)\phi \left(x_{1}\right)}{h}+\left[a\left(x_{3}\right)\left(\alpha nr\left(x_{3}\right)-2mx_{3}-n\right)-e\left(x_{3}\right)\left(x_{3}-h_{0}\right)\;-\mu \left(x_{3}\right)\right]\frac{d^{2}w}{dx_{1}^{2}}$$

Hence, the electric Gibbs free energy density is obtained in terms of the deflection $w\left(x_{1}\right)$ and the surface electric potential $\phi \left(x_{1}\right)$. Substituting Equations (12), (13), (22)–(25) into Equation (7), we can obtain the electric Gibbs free energy density

(26) $$U=\frac{1}{2}g\left(x_{3}\right){\left(\frac{d^{2}w}{dx_{1}^{2}}\right)}^{2}+i\left(x_{3}\right)\frac{\phi \left(x_{1}\right)}{h}\frac{d^{2}w}{dx_{1}^{2}}-\alpha^{2}a\left(x_{3}\right)r^{2}\left(x_{3}\right)\frac{\phi^{2}\left(x_{1}\right)}{2h^{2}}$$

where

(27) $$\begin{array}{ll} g\left(x_{3}\right)=[c\left(x_{3}\right) & -4me\left(x_{3}\right)-4m^{2}a\left(x_{3}\right)]x_{3}^{2}\\ & -\left[\left(2h_{0}c\left(x_{3}\right)+4mh_{0}e(x_{3}\right)+2ne(x_{3}\right)+4mna\left(x_{3}\right)+4m\mu \left(x_{3}\right)\\ & -2\alpha ne\left(x_{3}\right)r\left(x_{3}\right)-2\alpha mna\left(x_{3}\right)r\left(x_{3}\right)]x_{3}+c\left(x_{3}\right)h_{0}^{2}\\ & -2\alpha nh_{0}e\left(x_{3}\right)r\left(x_{3}\right)+2nh_{0}e\left(x_{3}\right)+\alpha n\mu \left(x_{3}\right)r\left(x_{3}\right)-n\mu \left(x_{3}\right)\\ & -\alpha^{2}n^{2}a\left(x_{3}\right)r^{2}\left(x_{3}\right)+n^{2}a\left(x_{3}\right)+2\alpha n^{2}a\left(x_{3}\right)r\left(x_{3}\right) \end{array}$$

(28) $$i\left(x_{3}\right)=\alpha r\left(x_{3}\right)\left[e\left(x_{3}\right)\left(x_{3}-h_{0}\right)+\mu \left(x_{3}\right)-\left(\alpha nr\left(x_{3}\right)-2mx_{3}-n\right)\right]\;$$

In order to find the governing equation and the corresponding boundary conditions, the Gibbs free energy density function of the graded beam is rewritten by the variation method

(29) $$\begin{array}{ll} \delta \int_{v}Udv=b\int_{-\frac{h}{2}}^{\frac{h}{2}} & \int_{0}^{L}\delta Udx_{1}dx_{3}\\ & =\frac{b}{2}\int_{-\frac{h}{2}}^{\frac{h}{2}}\int_{0}^{L}\{g\left(x_{3}\right)\delta {\left(\frac{d^{2}w}{dx_{1}^{2}}\right)}^{2}+2i\left(x_{3}\right)\delta \left[\frac{\phi \left(x_{1}\right)}{h}\frac{d^{2}w}{dx_{1}^{2}}\right]-\alpha^{2}a\left(x_{3}\right)r^{2}\left(x_{3}\right)\delta \left[\frac{\phi^{2}\left(x_{1}\right)}{h^{2}}\right]\}dx_{1}dx_{3} \end{array}$$

According to the generalized variational principle, we have δH=0, i.e.,

(30) $$\delta H=\int_{0}^{L}[Y_{ep}\frac{d^{4}w}{dx_{1}^{4}}-q\left(x_{1}\right)\delta w+\left(c_{pf}\frac{d^{2}w}{dx_{1}^{2}}-\frac{Qb}{h^{2}}\phi +\varpi b\right)\delta \phi ]dx_{1}+\left(Y_{ep}\frac{d^{2}w}{dx_{1}^{2}}+c_{pf}\phi \right)\delta \frac{dw}{dx_{1}}|\begin{array}{c} L\\ 0 \end{array}\;-\;\;\;\;\;\;\;\;\;\;\;\;\;\;\;\;\;Y_{ep}\frac{d^{3}w}{dx_{1}^{3}}\delta w|\begin{array}{c} L\\ 0 \end{array}=0$$

(31) $$Y_{ep}=b\int_{-\frac{h}{2}}^{\frac{h}{2}}g\left(x_{3}\right)dx_{3}$$

(32) $$c_{pf}=\frac{b}{h}\int_{-\frac{h}{2}}^{\frac{h}{2}}i(x_{3})dx_{3}$$

(33) $$Q=\alpha^{2}\int_{-\frac{h}{2}}^{\frac{h}{2}}a\left(x_{3}\right)r^{2}\left(x_{3}\right)dx_{3}\;$$

where $Y_{ep}$ is the effective bending rigidity of the FGM flexo-piezoelectric beam, which is related to elastic coefficient, piezoelectric coefficient, dielectric coefficient, and flexoelectric coefficient due to the gradient distribution of the materials. However, the flexoelectric coefficient has no influence on the effective bending rigidity of the monolayer or multi-layer nanobeams [10,11]. $c_{pf}$ is defined as the flexo-piezoelectric coupling parameter of the FGM flexo-piezoelectric beam. For piezoelectric beams ($\mu \left(x_{3}\right)=0$), the gradient distribution of the piezoelectric coefficient could also generate the piezoelectric mimicry of flexoelectricity [24].

For actuator structures, the fixed external electric voltage is loaded on the surface electrodes of the beams, which is called the CCF electrical condition boundary [10]. For sensor structures, there exists an induced electric potential by the mechanical deformation on the surface electrodes, which is called the OCI electrical condition boundary [10]. In the CCF condition, $\phi \left(x_{1}\right)$ is a constant $V_{0}$ and independent on the mechanical load. Due to the arbitrariness of δw, we have

(34) $$\{\begin{array}{c} Y_{ep}\frac{d^{4}w}{dx_{1}^{4}}-q\left(x_{1}\right)=0,\;0<x_{1}<L\\ w=\frac{dw}{dx_{1}}=0,x_{1}=0\\ Y_{ep}\frac{d^{2}w}{dx_{1}^{2}}+c_{pf}V_{0}=0,\frac{d^{3}w}{dx_{1}^{3}}=0,x_{1}=L \end{array}$$

Solving the equilibrium equation with corresponding boundary conditions, the deflection of the FGM actuator is

(35) $$w=\frac{q}{Y_{ep}}\left(\frac{x_{1}^{4}}{24}-\frac{x_{1}^{3}L}{6}+\frac{x_{1}^{2}L^{2}}{4}\right)-\frac{c_{pf}V_{0}}{2Y_{ep}}x_{1}^{2}$$

where the uniform lateral force q has been used for simplicity. Equation (35) clearly shows that the flexo-piezoelectric coupling parameter and external voltage on the electrodes (e.g., supplied by a battery) have significant effects on the mechanical responses of FGM actuators.

In the OCI condition, only distributed mechanical loads are applied on the surface of the FGM beam. The induced electric potential will be generated due to the electro-mechanical coupling, which is independent on $x_{1}$ and dependent on the mechanical load. Due to the arbitrariness of δw and δϕ, we have

(36) $$\{\begin{array}{c} Y_{ep}\frac{d^{4}w}{dx_{1}^{4}}-q\left(x_{1}\right)=0,\;0<x_{1}<L\\ \int_{0}^{L}\left(c_{pf}\frac{d^{2}w}{dx_{1}^{2}}-\frac{Qb}{h^{2}}\phi +\varpi b\right)dx_{1}=0\\ w=\frac{dw}{dx_{1}}=0,\;x_{1}=0\\ Y_{ep}\frac{d^{2}w}{dx_{1}^{2}}+c_{pf}\phi =0,\;\;\frac{d^{3}w}{dx_{1}^{3}}=0,\;x_{1}=L \end{array}$$

Under the open circuit condition, $\int_{0}^{L}\varpi bdx_{1}=0$ (no supply of charges to the electrodes) in Equation (36) and thus

(37) $$\phi =\frac{c_{pf}h^{2}}{bLQ}\frac{dw}{dx_{1}}{|}_{x_{1}=L}$$

Substituting Equation (37) into the boundary condition of Equation (36) and solving the equilibrium equation, we can obtain the deflection of FGM sensors as

(38) $$w=\frac{q}{Y_{ep}}\left[\left(\frac{x_{1}^{4}}{24}-\frac{x_{1}^{3}L}{6}+\frac{x_{1}^{2}L^{2}}{4}\right)-\frac{c_{pf}^{2}h^{2}}{12\left(c_{pf}^{2}h^{2}+Y_{ep}Qb\right)}x_{1}^{2}L^{2}\right]$$

Substituting Equation (38) into Equation (37), the induced electric potential of the FGM cantilever nanobeam can be obtained.

## 4. Numerical Results and Discussion

In this section, we perform simulations about the FGM nanobeam exhibiting the uniform lateral force or external voltage on the surface electrodes. In the present numerical example, BaTiO_3_ and PVDF are chosen. In order to keep the gradient index α the same in Equation (11), the electrical properties of PVDF and BaTiO_3_ are as follows: $a_{P}=1.248nC/\left(V\cdot m\right)$*,* $e_{P}=-0.44C/m^{2}$*,* $\mu_{P}=10^{-7}C/m$*,* $a_{B}=12.48nC/\left(V\cdot m\right)$*,* $e_{B}=-4.4C/m^{2}$*,* and $\mu_{B}=10^{-6}C/m$. The elastic properties of PVDF and BaTiO_3_ are as follows: $c_{P}=3.7GP$ and $c_{B}=131GPa$*,* respectively [29,30,31]. From the above parameters, we can obtain the corresponding gradient index parameters, i.e., α=−2.30 and $\alpha_{1}=-3.57$. When the gradient index infinitely approaches zero, the FGM beam will become a homogenous beam. In all numerical cases, the cross-sectional shape is kept the same by setting L=20h,and b=h. In the following numerical case, BTO-PVDF beams mean that the bottom surface is BaTiO_3_-rich and the top surface is PVDF-rich, and PVDF-BTO beams mean that the bottom surface is PVDF-rich and the top surface is BaTiO_3_-rich.

### 4.1. Closed Circuit with a Fixed External Electric Potential (CCF)

**Figure 2:**
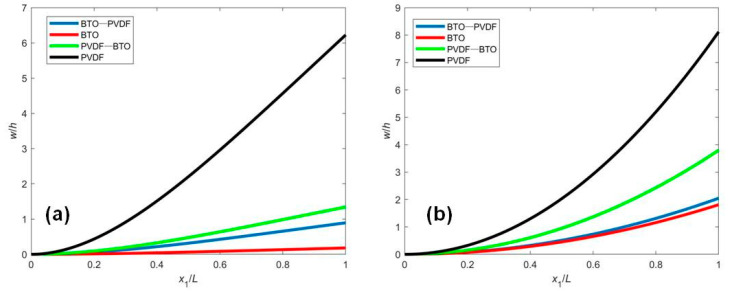
The normalized deflection w/h of FGM piezoelectric cantilever nanobeam subjected to (**a**) mechanical loading q=0.01nN/nm, $V_{0}=0$ and (**b**) electrical loading $V_{0}=-1V$, q=0.

The normalized deflection w/h of the FGM nanobeam with four different distributions of materials is plotted in Figure 2, in which only mechanical loading or electrical loading has been applied. It is observed from Figure 2 that the deflections of BTO and PVDF beams are the smallest and largest, respectively, and the deflection of BTO-PVDF beam is smaller than that of PVDF-BTO beams. It can be explained that the different polarization directions make the effective bending rigidity and flexo-piezoelectric coupling parameter different. By comparing the results of Figure 2a,b, the responses of the deflection induced by applied mechanical and electrical loading are similar.

**Figure 3:**
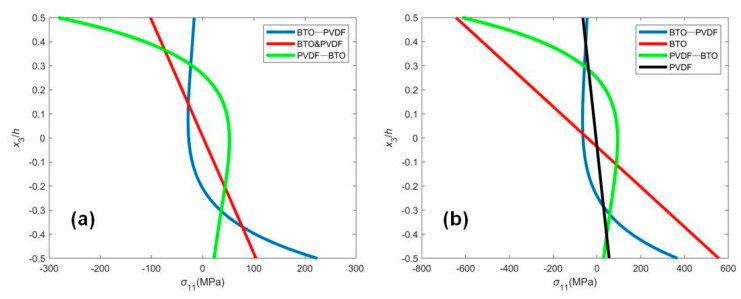
The stress distribution $\sigma_{11}$ of the FGM piezoelectric cantilever nanobeam subjected to (**a**) mechanical loading q=0.01nN/nm, $V_{0}=0$ and (**b**) electrical loading $V_{0}=-1V$, q=0.

Figure 3 illustrates the internal stress distribution at the fixed end with mechanical loading or electrical loading for four different distributions of materials. Figure 3 shows that the stress distribution of the uniform beam is linear, and the maximum stress is on the top and bottom surfaces. However, the stress distribution of the FGM cantilever nanobeams varies nonlinearly along the thickness, dependent on the gradient. In the mechanical loading case, there exists the same stress distribution in BTO and PVDF beams. However, in the electrical loading case, the stress on the surface in the BTO beam is larger than that in the PVDF beam since the elastic stiffness of the BTO beam is greater. It is observed from Figure 3 that the largest compressive stress in the BTO-PVDF beam and the largest tensile stress in the PVDF-BTO beam occur in the interior of the functionally graded beam, which is in agreement with the previous results [22].

### 4.2. Open Circuit with Surface Electrodes and an Induced Electric Potential by Mechanical Deformation (OCI)

**Figure 4:**
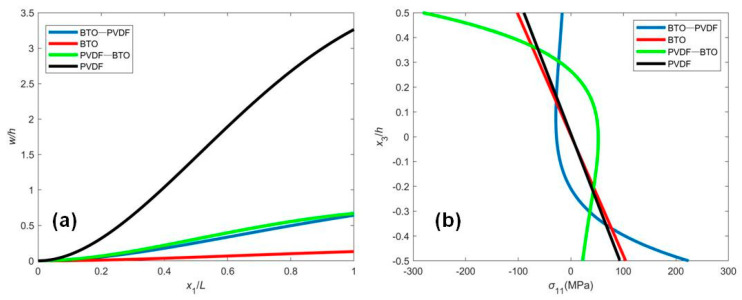
(**a**) The normalized deflection w/h and (**b**) stress distribution $\sigma_{11}$ of FGM sensors subjected to the mechanical loading q=0.01nN/nm.

Figure 4 shows the normalized deflection and stress distribution of the FGM beam with the induced electric potential under the OCI condition. In this case, the induced electric potential is generated by the mechanical deformation due to the flexoelectric effect. The deflection is smaller, and the shape of the deflection curve is different from the results in Figure 2a. Apparently, the converse flexoelectricity induces a uniform electric field in the beam, which in turn induces a uniform bending moment along the beam acting in the direction against the mechanical load. As a result, the deflection is reduced by the flexoelectric effect. From Figure 4b, we can also observe that the stress distribution, in this case, is smaller than that in Figure 3.

**Figure 5:**
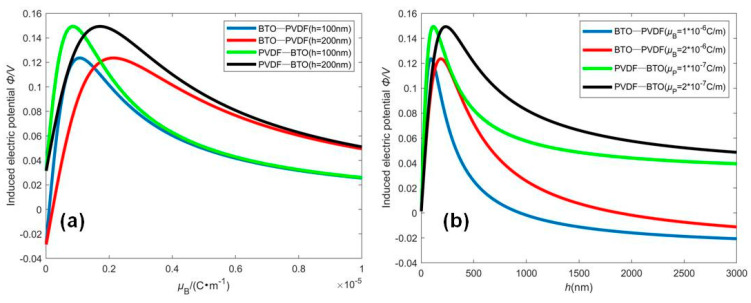
The variation of the induced electric potential of FGM sensors subjected to the mechanical loading q=0.01nN/nm vs. (**a**) the flexoelectric coefficient and (**b**) the beam thickness.

The induced electric potential due to the flexoelectric effect, which has a significant scaling effect, is very important for sensors and energy harvesters. Figure 5 is presented to investigate the variation of the induced electric potential with respect to the flexoelectric coefficient and beam thickness. Since bending a homogeneous piezoelectric beam cannot give a flexoelectriclike response, the induced electric potential of BTO and PVDF beams is not given in order to investigate the piezoelectric gradient. The piezoelectric coefficient asymmetrically distributed across the beam thickness will generate an induced electric potential subjected to bending deformation. With fixed cross-sectional shape and L/h, the maximum induced electric potential is independent of the flexoelectric coefficient or the beam thickness for the same material distribution, which are similar to that of homogeneous flexo-piezoelectric nanobeams [10,11]. However, for the large flexoelectric coefficient, the maximum induced electric potential occurs due to the large beam thickness. From Figure 5, it is observed that the maximum induced electric potential of the PVDF-BTO graded beam is greater than that of the BTO-PVDF graded beam. It can be easily explained that the sign of the piezoelectric coefficient or the polarization direction of the FGM beam has a very significant influence on the induced electric potential [11,24]. The piezoelectricity would change significantly under space inversion, but the elastic, flexoelectric, and dielectric properties are insensitive to space inversion. Hence, the PVDF-BTO and BTO-PVDF graded beams have the same elastic, flexoelectric, and dielectric distributions but the opposite polarization direction. Without the flexoelectric effect or with a large thickness, the PVDF-BTO and BTO-PVDF graded beams have positive or negative induced electric potential, respectively, as shown in Figure 5. The results in Figure 5 also illustrate that the effective flexoelectric effect caused by nonuniform piezoelectricity is functionally identical to intrinsic flexoelectricity. Hence, we can gain an optimal electrical performance of sensors or energy harvesters by changing the polarization distribution and thickness of the beams. Figure 5b also displays that the flexoelectricity has a significant effect at a small scale and that the piezoelectricity plays an important role at a larger scale. The present conclusions can be used to obtain the optimal electrical output for designing functionally graded structures.

## 5. Discussion

The electro-mechanical analysis of an FGM piezoelectric beam with flexoelectricity is investigated in the present paper. Based on the electric Gibbs free energy and the linear piezoelectric theory, the generalized variational principle is applied to derive the governing equation and the corresponding boundary conditions under the CCF and OCI conditions. Some new coupling parameters are proposed to describe the interplay between piezoelectricity and flexoelectricity. The analytical expressions of the deflection and induced electric potential are given for the static bending problem. The numerical results reveal that the deflection, stress distribution, and induced electric potential are dependent on the flexoelectric effect and gradient distribution. Nonuniform piezoelectricity along the thickness will generate remarkable effective flexoelectricity, which is very important for large-scale structures. Moreover, the flexoelectric effect can significantly enhance the electrical performance of FGM sensors at the nanoscale. In general, the coupling effect of FGM nanobeams will be different from that of homogeneous beams, and reasonable gradient distribution will enhance the electro-mechanical coupling performance. The present result could be helpful in understanding the distribution design of composite materials.

## Data Availability

Data are available upon request from the corresponding author.
